# Perilesional and homotopic area activation during proverb comprehension after stroke

**DOI:** 10.1002/brb3.1202

**Published:** 2018-12-26

**Authors:** You Gyoung Yi, Dae Yul Kim, Woo Hyun Shim, Joo Young Oh, Ho Sung Kim, Minji Jung

**Affiliations:** ^1^ Department of Rehabilitation Medicine, Seoul National University Hospital Seoul National University College of Medicine Seoul Korea; ^2^ Department of Rehabilitation Medicine Asan Medical Center Seoul Korea; ^3^ Department of Radiology and Research Institute of Radiology Asan Medical Center Seoul Korea; ^4^ Asan Institute for Life Science, Asan Medical Center University of Ulsan College of Medicine Seoul Korea

**Keywords:** functional MRI, language, neural inhibition, proverb, right middle cerebral artery infarction

## Abstract

**Introduction:**

The mechanism of functional recovery in right hemisphere (RH) stroke patients when attempting to comprehend a proverb has not been identified. We previously reported that there is bilateral hemisphere involvement during proverb comprehension in the normal population. However, the underlying mechanisms of proverb comprehension following a right middle cerebral artery (MCA) infarction have not yet been fully elucidated.

**Methods:**

We here compared the brain regions activated by literal sentences and by opaque or transparent proverbs in right MCA infarction patients using functional magnetic resonance imaging (fMRI). Experimental stimuli included 18 opaque proverbs, 18 transparent proverbs, and 18 literal sentences that were presented pseudorandomly in 1 of 3 predesigned sequences.

**Results:**

Fifteen normal adults and 17 right MCA infarction patients participated in this study. The areas of the brain in the stroke patients involved in understanding a proverb compared with a literal sentence included the right middle frontal gyrus (MFG) and frontal pole, right anterior cingulate gyrus/paracingulate gyrus and left inferior frontal gyrus (IFG), middle temporal gyrus (MTG), precuneus, and supramarginal gyrus (SMG). When the proverbs were presented to these stroke patients in the comprehension tests, the left supramarginal, postcentral gyrus, and right paracingulate gyrus were activated for the opaque proverbs compared to the transparent proverbs.

**Conclusions:**

These findings suggest that the functional recovery of language in stroke patients can be explained by perilesional activation, which is thought to arise from the regulation of the excitatory and inhibitory neurotransmitter system, and by homotopic area activation which has been characterized by decreased transcallosal inhibition and astrocyte involvement.

## 1 INTRODUCTION

There has been considerable debate on whether the right hemisphere (RH) is involved in understanding figurative language in normal subjects (Bookheimer, 2002; Bottini et al., 1994; Diaz, Barrett, & Hogstrom, 2011; Faust & Mashal, 2007; Kana, Murdaugh, Wolfe, & Kumar, 2012; Lai, Dam, Conant, Binder, & Desai, 2015; Lee & Dapretto, 2006; Mashal, Faust, & Hendler, 2005; Mitchell, Vidaki, & Lavidor, 2016; Oliveri, Romero, & Papagno, 2004; Papagno, Curti, Rizzo, Crippa, & Colombo, 2006; Proverbio, Crotti, Zani, & Adorni, 2009; Yang et al., 2016; Zempleni, Haverkort, Renken, & Stowe, 2007). It has recently been shown that RH activation can be influenced by various factors such as conventionality at either a word‐level or in a sentential context, by task type, and by the level of transparency (Anaki, Faust, & Kravetz, 1998; Diaz et al., 2011; Faust & Chiarello, 1998; Faust & Mashal, 2007; Mashal et al., 2005; Rapp, Mutschler, & Erb, 2012; Schmidt & Seger, 2009; Sela, Panzer, & Lavidor, 2017; Yang et al., 2016). It has been demonstrated previously that metaphoric comprehension involves an enhanced role of the right hemisphere in accordance with hemispheric semantic processing (Anaki et al., 1998). The right insula, left temporal pole, and right inferior frontal gyrus have been reported to be activated for metaphor processing, in comparison with literal sentences (Schmidt & Seger, 2009). However, Oliveri et al. (2004) have reported that the understanding of opaque idioms is closely related to the left hemisphere and suggested that the superior temporal cortex is crucially involved in the process of understanding idioms. Recently also, it has been reported that the bihemispheric network is involved in idiom comprehension, and that hemispheric asymmetry is affected by idiom predictability (Sela et al., 2017).

In our previous preliminary study in normal Korean subjects, we identified using functional magnetic resonance imaging (fMRI) that the left hemisphere, including the left inferior frontal gyrus (IFG), is more activated by a proverb than by a literal sentence (Yi et al., 2017). Additionally, we reported in that same study that the right precuneus and the right supramarginal gyrus (SMG) are activated when attempting to understand an opaque proverb, suggesting that the right hemisphere is involved in abstract language processing (Yi et al., 2017). There have been few comparable reports in brain‐injured patients however. There have been some studies on the mechanism of understanding language in left hemispheric stroke patients with aphasia with findings of RH homolog activation during language processing (Bartolomeo & Thiebaut de Schotten, 2016; Lidzba, Haan, Wilke, Krageloh‐Mann, & Staudt, 2017; Skipper‐Kallal, Lacey, Xing, & Turkeltaub, 2017; Turkeltaub, Messing, Norise, & Hamilton, 2011). In another study of proverb interpretation tasks (PIT) in frontal lobe‐injured patients, bilateral medial frontal lobe lesions were found to be associated with impaired performance (Murphy et al., 2013).

Notably however, the activation pattern in RH stroke patients who are attempting to understand a proverb has not been identified, nor whether this differs from the normal population. In a recent study of the role of the right hemisphere in semantic control, there was no significant difference found between RH‐damaged patients and normal subjects when they performed a standard battery of semantic tasks including a synonym judgment task (Thompson, Henshall, & Jefferies, 2016). This suggests that either the RH is not strongly associated with semantic tasks or that a compensatory mechanism exists in another part of the brain.

In our current study, we used fMRI to investigate the mechanisms by which right middle cerebral artery (MCA) infarction patients understand proverbs. Our hypothesis was that there would be more active areas in the brains of these patients than in normal people when attempting to understand the proverb. We also speculated that another part of the brain would be activated and involved in abstract processing in these stroke patients than the right SMG and precuneus that is seen in normal subjects.

## 2 METHODS

### 2.1 Participants

Fifteen normal adults and 17 right MCA infarction patients were enrolled from March 2014 to November 2015. The inclusion criteria for both normal adults and stroke patients were an age of 19 years or older and a four‐year college education. The specific inclusion criteria for the stroke patients were as follows: native Korean speaker, right handed by self‐reporting, and a Mini‐Mental State Examination (MMSE) ≥24 after the stroke. We excluded stroke patients who showed a contraindication for fMRI (magnetic stimulation not allowable due to a metallic implant, artificial pacemaker, cochlear implant, or cerebral metal insertion), suffered from claustrophobia, apraxia, aphasia, or neglect, declined to participate, had any history or risk of seizure, or had other neurologic diseases.

### 2.2 Compliance with ethical standards

All experiments performed in the study were conducted in accordance with the ethical standards of the institutional and/or national research committee and with the 1964 Helsinki declaration and its later amendments or comparable ethical standards. All participants provided written informed consent and the Asan Medical Center Institutional Review Board (IRB) approved the study design prior to participation (IRB No. 2014‐0261).

### 2.3 Types of figurative language

We categorized the proverbs used to test the participants by familiarity and opacity/transparency as indicated below.

#### 2.3.1 Familiarity

For an objective measurement of the familiarity of each stimulus, 200 proverbs (100 transparent proverbs and 100 opaque proverbs) were categorized by 100 native Korean speakers who did not take part in the imaging experiment to evaluate familiarity on a scale from 1 to 5 (1 = never used or encountered, 5 = frequently used or encountered). The 54 proverbs that were eventually selected for the analysis in each group (Table 1, reproduced with permission from Yi et al. 2017) were scored similarly as follows: the transparent proverbs had a mean familiarity score of 4.13 (standard deviation [*SD*] = 0.62) and the opaque proverbs had a mean familiarity score of 4.04 (*SD* = 0.47).

**Table 1:** Characteristics of the experimental stimuli (reproduced with permission from Yi et al., 2017)

Stimulus	Semantic distance	Proverb status	Familiarity	Number of Korean characters
Opaque proverbs	5.02 (0.63)	1 (0)	4.04 (0.47)	11.19 (2.63)
Transparent proverbs	2.33 (0.48)	1 (0)	4.13 (0.62)	11.26 (2.47)
Literal sentences	0.02 (0.14)	0 (0)	4.20 (0.56)	11.15 (2.48)

#### 2.3.2 Transparency

The stimuli used in our current study were those used in our previous preliminary study (Yi et al., 2017). Briefly, we compared opaque and transparent proverbs to identify the effects of transparency processing during proverb comprehension. Examples of opaque and transparent proverbs are demonstrated in Table 2. Two speech‐language pathologists, a scholar of Korean literature, and five healthy adults who did not take part in the imaging experiment categorized the degree of semantic distance with 200 proverbs using scores ranging from 1 to 7 (1 = very close, 7 = very distant) for an objective measurement of the transparency of each stimulus.

**Table 2:** Example of a literal sentence, opaque proverb and transparent proverb (reproduced with permission from Yi et al., 2017)

Classification	Korean	Alphabet sound	Meaning
Literal sentence	마라톤 선수가 결승점에 도착했다	Ma/la/ton seon/su/ga gyeol/seung/jeom/e do/chag/haess/da	The marathon runner has arrived at the finish line
Opaque proverb	병에 찬 물은 저어도 소리가 안 난다	Byeong/e chan mul/eun jeo/eo/do so/li/ga an nan/da	Bottled water has no sound even when poured. Hidden meaning: A person who actually knows a lot is humble
Transparent proverb	가는 말이 고와야 오는 말이 곱다	Ga/neun mal/i go/wa/ya o/neun mal/i gob/da	If you say nice things, nice things will be said to you.

### 2.4 Stimuli

**Figure 1:**
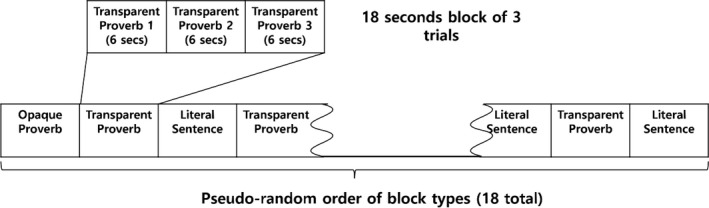
Experimental Design. (reproduced with permission from Yi et al., 2017). In each trial, the sentence (both proverbs and literal sentences) appeared in the center of the screen for 6 s. The 54 sentences used were randomly assigned to an 18‐s block containing three sentences of the same type arranged in a pseudorandom order. A total of 18 blocks composed of six blocks of transparent figurative proverbs, six blocks of opaque proverbs, and six blocks of literal sentences

Fifty‐four opaque proverbs (1), 54 transparent proverbs (2), and 54 literal sentences (3) were selected as the final experimental stimuli for the study subjects (Table 1, reproduced with permission from Yi et al., 2017). A total of 36 proverbs and 18 literal sentences were pseudorandomly presented to each subject in one of three predesigned sequences (Figure 1, reproduced with permission from Yi et al., 2017). All sentences were displayed on the screen while the subject was lying on the MRI scanner. The activated areas in response to the different stimuli were compared as follows: (a) literal sentences with proverbs and (b) transparent with opaque proverbs.

### 2.5 MRI data acquisition

Magnetic resonance imaging data were obtained using a 3 T system (Achieva; Philips Medical Systems, Best) equipped with an 8‐channel sensitivity‐encoding head coil. Functional images were obtained using blood oxygen level‐dependent (BOLD) contrast images by single‐shot gradient‐echo/echo‐planar imaging (GE/EPI) (repetition time/echo time (TR/TE) = 3,000/35 ms, flip angle = 90°, field of view = 220 mm, matrix = 128 × 128, slice thickness = 4 mm with no gap). A 3D gradient‐echo T1‐weighted sequence (TR/TE = 9.9/4.6 ms, flip angle = 8°, field of view = 224 mm, matrix = 448 × 448, slice thickness = 1 mm with no gap) was used to acquire the high‐resolution anatomical three‐dimensional (3D) volume image. The sentences were presented visually using a rear projection onto the screen mounted inside the bore. Sentences were displayed through a mirror mounted on the head coil. To allow for steady‐stage magnetization, the initial four dummy scans were discarded prior to the fMRI procedure. E‐Prime software (Psychology Software Tools, Pittsburgh, PA) was used to create all of the design and control stimuli in the scanner.

### 2.6 Data analysis

Processing of the fMRI was performed using the FSL 5.0.6 software package (Oxford Centre for Functional Magnetic Resonance Imaging of the Brain [FMRIB] Analysis Group, RRID = SCR_002823, www.fmrib.ox.ac.uk/fsl). FMRIB's Linear Image Registration Tool (MCFLIRT) was used to correct the head motions. All images were smoothed using a Gaussian kernel of 5‐mm full‐width half maximum. Brain Extraction Tool (BET) was used for skull stripping and nonbrain tissue removal. The imaging data were analyzed using FEAT (fMRI Expert Analysis Tool) general linear modeling with both the lower‐ and higher‐level modes. To register in the standard space (MNI‐152 space), all fMRI data were registered in each participant's anatomical T1 volumes. In all cases, the transformations were performed using a 12 Degrees Of Freedom (DOF) linear transformation. Statistically based parametric images were generated using the uncorrected significance threshold of *p* < 0.001. According to Badre & D'Esposito (Badre & D'Esposito, 2007), this value corresponds to a threshold *T*‐value of 4.5, which is commonly used in fMRI studies.

## 3 RESULTS

### 3.1 Baseline characteristics of the study participants

Normal subjects (eight women and seven men) were recruited with a mean age of 30.2 years (range, 27–33 years). The baseline characteristics of the right MCA infarction patients included in the study group are presented in Table 3.

**Table 3:** Baseline characteristics and computerized neuropsychological testing of the right MCA infarction patients (*n* = 17)

	Right MCA infraction patients (*n* = 17)
Male:Female (*n*)	9:8
Age (years)	38.4 (20–49)
K‐MMSE (score)	27.2 ± 3.3
Duration of stroke (day)	28.9 ± 11.9
K‐MBI (score)	51.5 ± 11.6
Attention	
VCPT (sec)	0.3 ± 0.2
ACPT (sec)	0.4 ± 0.1
DSFT	6.2 ± 1.1
DSBT	5.5 ± 0.9
VSFT	5.8 ± 1.0
VSBT	4.9 ± 1.3
Memory	
AVLT	
1st trial	12.6 ± 2.0
Delayed trial	11.3 ± 1.8
VRT	
1st trial	11.4 ± 1.1
Delayed trial	8.2 ± 1.4

Note. Values are indicated as a mean ± standard deviation.

ACPT: auditory continuous performance test; AVLT: auditory verbal learning test; DSBT: digit span backward test; DSFT: digit span forward test; MCA: middle cerebral artery; VCPT: visual continuous performance test; VRT: visual recognition test.; VSBT: visual span backward test; VSFT: visual span forward test.

Highest possible score in the DSFT, DSBT, VSFT, and VSBT is 8; for the AVLT and VRT this is 15.

### 3.2 Behavioral data

The tasks for each study subject were to attempt to comprehend the sentences and to verbally report on whether each sentence had been fully understood after the fMRI scan. For this purpose, all subjects were asked to explain the meaning of each literal sentence and proverb to confirm whether they understood them correctly. The right MCA infarction cases were also evaluated using a computerized neuropsychological test (CNT) which included visual and auditory continuous performance tests, forward and backward digit span tests, forward and backward visual span tests, and visual and verbal learning tests (Table 3).

### 3.3 Understanding of proverbs versus literal sentences in the right MCA infarction group

**Figure 2:**
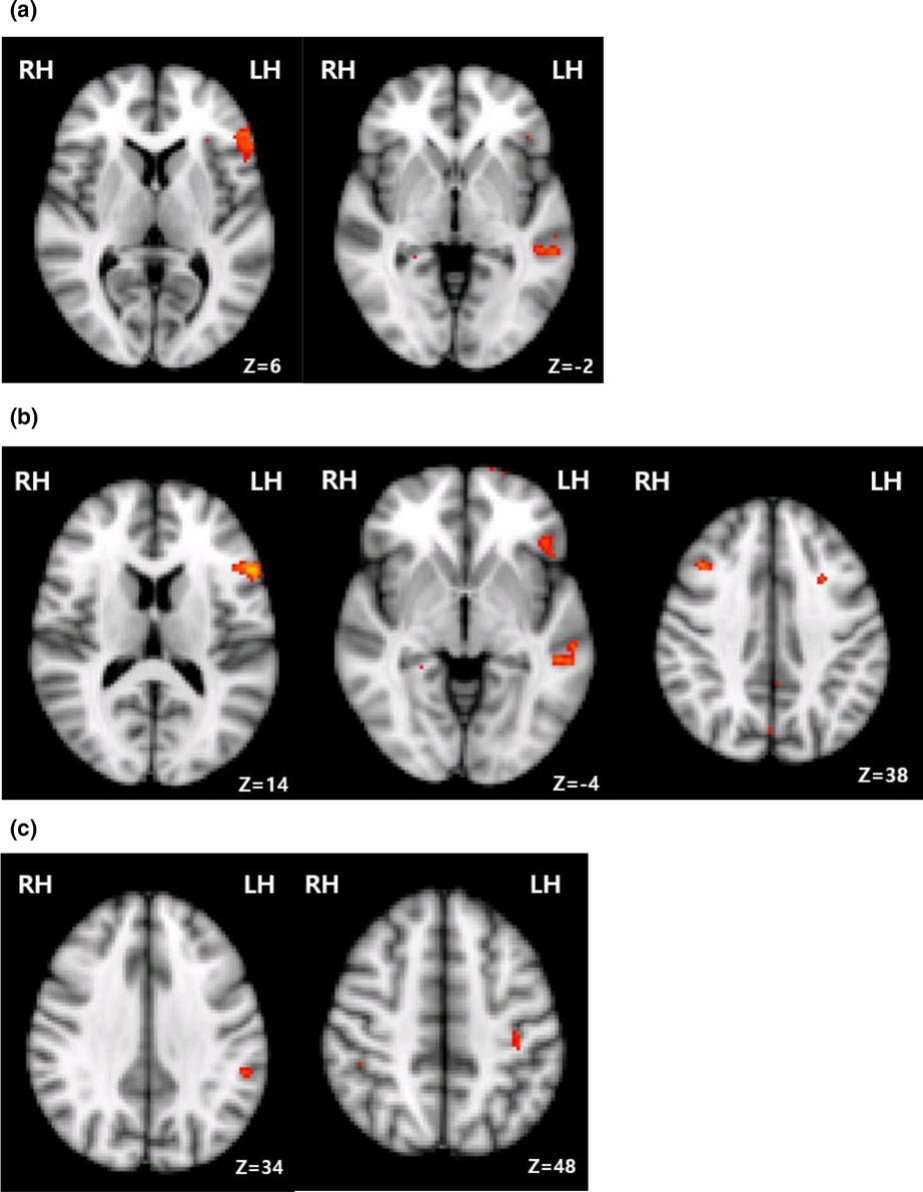
Activated areas of the brain during proverb comprehension compared with that for literal sentences in the right MCA infarction patients. The images show regions of significant differences in activation (*p* < 0.001, uncorrected [T ≥ 3.30]). The spatial extent of that activation (kE) was ≥10 voxels. (a) Proverb > literal sentence in right MCA infarction patients (b) Proverb > literal sentence, right MCA infarction patients > normal subjects (c) Opaque proverb > transparent proverb in right MCA infarction patients. H: hemisphere; L: left; R: right

The left hemisphere including the left IFG showed overall activation in the right MCA infarction patients during proverb comprehension as compared to reading literal sentences (Table 4, Figure 2), and the left middle temporal gyrus (MTG) and the SMG were significantly activated. The middle frontal gyrus (MFG), inferior temporal gyrus, and frontal pole in the right hemisphere in the stroke patients were activated during these tasks (Table 4). When compared with the proverb comprehension test, we found that literal sentence comprehension activated the left parietal operculum cortex and the right cingulate gyrus.

**Table 4:** Brain regions in the right MCA infarction patients showing contrast significance during the comprehension tests (*p* < 0.001)

kE	T	Lat	Anatomical region		x	y	z	BA
Proverb > Literal sentence								
305	3.80	LH	Inferior frontal gyrus	−52		26	6	45
71	3.79			−56		34	6	45
90	3.46		Frontal orbital cortex	−42		32	−12	47
58	3.39		Middle temporal gyrus	−54		−36	−2	21
22	3.33		Supramarginal gyrus	−54		−38	34	40
11	3.35		Precuneus	−8		−62	42	7
24	3.49	RH	Inferior temporal gyrus	48		−56	−10	37
14	3.76			50		36	20	9
23	3.57		Middle frontal gyrus	40		22	38	8
20	3.37		Frontal pole	38		56	−4	10
14	3.44		Paracingulate gyrus	6		36	30	32
Literal sentence > Proverb								
15	3.34	LH	Parietal operculum cortex	−54		−26	14	40
33	3.38	RH	Cingulate gyrus	0		−44	34	23

Note. All areas reported were significant at a p uncorrected ≤001 (T ≥ 3.30), and the spatial extent of activation (kE) was ≥10 voxels. Areas are presented with the stereotactic coordinates (x, y, z), the cytoarchitectural designation according to Brodmann (BA), the maximum T value (T) and the extent (kE) of the activated clusters. The hemispheric lateralization (Lat) is presented in a separate column to emphasize laterality effects.

H: hemisphere; L: left; R: right.

### 3.4 Comparison of right MCA infarction patients and normal subjects in understanding proverbs versus literal sentences

When compared with the activated regions in the normal subjects following the proverb comprehension testing, we found that the right middle frontal gyrus and frontal pole, right anterior cingulate gyrus/paracingulate gyrus and left IFG, MTG, precuneus, and SMG were more activated in the stroke patients (Table 5, Figure 2). We also found that the left SMG, MFG, and central opercular cortex were more activated in the stroke patients than in the normal control subjects following the opaque proverb comprehension testing, as were the left IFG and MTG following the transparent proverb comprehension.

**Table 5:** Brain regions showing contrast significance (*p* < 0.001) between the right MCA infarction patients and the normal subjects following the proverb comprehension tests

kE	T	Lat	Anatomical region	x	y	z	BA
Proverb > Literal sentence							
48	3.39	LH	Middle temporal gyrus	−54	−36	−4	21
36	3.30		Inferior frontal gyrus	−54	20	14	44
12	3.32		Supramarginal gyrus, anterior division	−56	−38	34	40
12	3.37		Precuneus	−7	−62	42	7
37	4.12	RH	Frontal pole	32	56	8	10
33	3.66		Middle frontal gyrus	40	22	38	8
33	3.28		Cingulate gyrus	0	−44	34	23
51	3.51		Paracingulate gyrus	6	36	30	32
Opaque proverb > Literal sentence							
22	3.31	LH	Supramarginal gyrus, anterior division	−54	−38	34	40
12	3.35		Central opercular cortex	−54	−10	18	4
23	3.31		Middle frontal gyrus	−44	14	30	44
Transparent proverb > Literal sentence							
21	3.34	LH	Inferior frontal gyrus	−56	34	6	45
15	3.41		Middle temporal gyrus	−60	−36	−4	21

Note. All areas reported were significant at a p uncorrected ≤001 (T ≥ 3.30), and the spatial extent of activation (kE) was ≥10 voxels. Areas are presented with the stereotactic coordinates (x, y, z), the cytoarchitectural designation according to Brodmann area (BA), the maximum T value (T) and the extent (kE) of the activated clusters. The hemispheric lateralization (Lat) is presented in a separate column to emphasize laterality effects.

H: hemisphere; L: left; R: right.

### 3.5 Comprehension of opaque versus transparent proverbs among the right MCA infarction patients

When the proverbs were presented to the stroke patients in the comprehension tests, the left supramarginal, postcentral gyrus, and right paracingulate gyrus were activated for the opaque proverbs compared to the transparent proverbs (Table 6, Figure 2). When compared with the opaque proverbs, transparent proverbs activated left superior frontal gyrus and MTG.

**Table 6:** Brain regions showing contrast significance (*p* < 0.001) during opaque versus transparent proverb comprehension in the right MCA infarction patients

kE	T	Lat	Anatomical region	x	y	z	BA
Opaque proverb > Transparent proverb							
15	3.32	LH	Middle frontal gyrus	−28	14	40	9
32	3.32		Supramarginal gyrus	−54	−38	34	40
13	3.34		Postcentral gyrus	−36	−26	48	1
12	3.34	RH	Paracingulate gyrus	6	36	32	32
Transparent > Opaque proverb							
24	3.48	LH	Superior frontal gyrus	−10	36	50	8
21	3.31		Middle temporal gyrus	−54	−34	−4	21

Note. All areas reported were significant at a p uncorrected ≤001 (T ≥ 3.30), and the spatial extent of activation (kE) was ≥10 voxels. Areas are presented with the stereotactic coordinates (x, y, z), the cytoarchitectural designation according to Brodmann area (BA), the maximum T value (T) and the extent (kE) of the activated clusters. The hemispheric lateralization (Lat) is presented in a separate column to emphasize laterality effects.

H: hemisphere; L: left; R: right.

## 4 DISCUSSION

### 4.1 Brain recruitment patterns involved in proverb comprehension in right MCA infarction patients

In both the right MCA infarction patients and normal control subjects recruited into our current study series, a robust activation in the left IFG was observed during proverb comprehension as compared with literal sentences. During the proverb test, activation in the right MCA infarction patients was higher than the normal subjects in the left IFG, MTG, and supramarginal gyrus, and in the right frontal pole, MFG, and paracingulate gyrus. Because our study cohort included patients who had undergone a right MCA infarction, we speculated that a neural substrate may be released from the right MFG and frontal pole near to the injured area during the comprehension test, resulting in perilesional activation. Indeed, perilesional activation has been shown to be the principal mechanism of motor recovery following a subacute stroke (Badre & D'Esposito, 2007). Moreover, Lidzba and colleagues have shown in their previous study that postfrontal “activation spots” can be observed after motor aphasia, which reflect the perilesional reorganization that would be involved in functional recovery (Gulati et al., 2015). As perilesional activation has been identified as an important mechanism of motor function recovery in stroke patients, attempts have been made recently to use intracortical brain–machine interfaces (BMI) for motor recovery in stroke patients (Gulati et al., 2015). Perilesional activation is thought to arise from the regulation of excitatory and inhibitory neurotransmitter systems in the cerebral cortex (Griffis, Nenert, Allendorfer, & Szaflarski, 2017; Hylin, Kerr, & Holden, 2017), which has not yet been fully established as the mechanism of language recovery. Our present study findings demonstrate that perilesional activation occurs during proverb comprehension in patients with a right MCA infarction.

The contralateral homologous cortex, such as the left MTG and IFG, may have been activated due to decreased transcallosal inhibition through the right hemisphere as a result of the MCA infarction (Badre & D'Esposito, 2007; Gulati et al., 2015; Skipper‐Kallal et al., 2017; Turkeltaub et al., 2011). Lidzba et al. reported previously that an activation pattern could be observed in homotopic right hemispheric lesions in patients with congenital white‐matter lesions in the left hemisphere language area (Lidzba et al., 2017). In another study of patients with left hemispheric brain damage after epilepsy surgery, activation patterns were observed in both the lesion and homotopic areas (Tivarus, Starling, Newport, & Langfitt, 2012). A previous study with transcranial magnetic stimulation and fMRI reported that the contralesional dorsal premotor cortex may support residual motor function following stroke (Bestmann et al., 2010). In our present study, we found that some of the homotopic left hemispheres involved in proverb comprehension were more activated in the stroke patients than in the normal subjects, which is consistent with the findings of previous studies. The mechanism of functional recovery contralateral to the lesion has been demonstrated to involve the astrocytes (Takatsuru et al., 2013). The authors of that same study found that an increase in extracellular glutamine levels was an indicator of astrocyte involvement and that astrocytes also played a role in the conversion of glutamate to glutamine, an important process for functional recovery. Although we did not quantitatively assess the degree of recovery of proverb comprehension in our current study series, we found that all of our study patients correctly comprehended the proverbs and had some degree of restored language function. An increase in brain activation indicated on an fMRI has been reported previously to be proportional to motor recovery (Ward, Brown, Thompson, & Frackowiak, 2006), demonstrating that functionally relevant changes in cerebral organization can be identified through the use of these images.

### 4.2 Abstract language processing in patients with right MCA infarction

It was previously reported that the semantic similarity effect for words is limited to the left perirhinal cortex (Bruffaerts et al., 2013). In our own previous study in a normal population of proverb comprehension, we reported that the right precuneus and right supramarginal gyrus are involved in abstract language processing (Yi et al., 2017). In our current study, we observed that transcallosal inhibition (Elkana, Frost, Kramer, Ben‐Bashat, & Schweiger, 2013; Griffis et al., 2017; Hartwigsen, 2016; Hartwigsen et al., 2010; Hylin et al., 2017; Lim & Kang, 2015) through the right hemisphere might be reduced because right hemisphere stroke patients show a persistent lesion for about 30 days even when the recovery mechanism has begun. The contralateral homologous cortex may be activated when these patients attempt to comprehend an opaque proverb that requires semantic processing. In addition, the activation of the right paracingulate gyrus could be part of the supracallosal medial frontal cortex involvement in speech and language generation and may compensate for the loss of function following a right MCA infraction. Although our present study involved right hemispheric stroke patients, this suggests that this region is involved in coordinated processing during disturbed semantic processing.

### 4.3 Limitations and future directions

Our present analyses had some limitations of note. The ages of the stroke patients and normal subjects were not matched and the mean age difference between these two groups was 8 years. In addition, although our experimental stimuli were controlled for familiarity, semantic distance and length, the word frequency was not matched. Moreover, opaque and transparent proverbs were matched for familiarity but not for length, although these two stimuli had almost identical lengths. We did not perform power analysis as it was difficult to recruit sufficient MCA infarction patients with the cognitive ability to understand proverbs. However, our cohort of 17 patients was larger than that analyzed in a comparable previous study (Tivarus et al., 2012). We only confirmed in our current study series whether the stroke patients understood the meaning of a proverb. We did not score their responses and therefore could not determine in this cohort whether perilesional activation contributed positively to functional recovery. However, the increase in brain activation evident on the fMRIs has been reported previously to be proportional to motor recovery (Ward et al., 2006). This demonstrated that functionally relevant changes in cerebral organization can be identified through fMRI alone. In addition, we performed our analysis during the subacute phase of the stroke, a time at which the opposite side of the infarcted lesion is relatively active. Since our fMRI analysis was not serialized, only the results from the subacute phase at which synaptogenesis is not yet complete were presented. Finally, It is also known that complex metaphors activate the left IFG (Schmidt & Seger, 2009) but the control of difficulty was not completely controlled in our present study.

## 5 CONCLUSION

The areas of the brain involved in proverb comprehension compared with literal sentences in right hemispheric stroke patients are the left IFG, left MTG, right MFG, and frontal pole. This activation pattern can be explained by the homotopic area and perilesional activation of the infarction area when compared with normal subjects. The areas involved in abstract language processing in right hemispheric stroke patients include the left SMG and left MFG, which is the homotopic area of that in normal population.

## DISCLOSURE

Dr. Dae Yul Kim is funded by the National Research Foundation of Korea (NRF). You Gyoung Yi, Woo Hyun Shim, Joo Young Oh, and Ho Sung Kim report no disclosures.

## CONFLICT OF INTEREST

None declared.
